# Forward Genetic Screen in *Caenorhabditis elegans* Suggests F57A10.2 and acp-4 As Suppressors of C9ORF72 Related Phenotypes

**DOI:** 10.3389/fnmol.2016.00113

**Published:** 2016-11-08

**Authors:** Xin Wang, Limin Hao, Taixiang Saur, Katelyn Joyal, Ying Zhao, Desheng Zhai, Jie Li, Mochtar Pribadi, Giovanni Coppola, Bruce M. Cohen, Edgar A. Buttner

**Affiliations:** ^1^School of Public Health, Xinxiang Medical UniversityXinxiang, China; ^2^Program for Neuropsychiatric Research, McLean Hospital and Harvard Medical SchoolBelmont, MA, USA; ^3^School of Pharmacy, Xinxiang Medical UniversityXinxiang, China; ^4^Tianjin Mental Health CenterTianjin, China; ^5^Semel Institute for Neuroscience and Human Behavior, David Geffen School of Medicine, University of California, Los AngelesLos Angeles, CA, USA

**Keywords:** *Caenorhabditis elegans*, C9ORF72, ALS-FTLD, VAPB, ACP2, amyotrophic lateral sclerosis

## Abstract

An abnormally expanded GGGGCC repeat in C9ORF72 is the most frequent causal mutation associated with amyotrophic lateral sclerosis (ALS)/frontotemporal lobar degeneration (FTLD). Both *gain-of-function* (*gf*) and *loss-of-function* (*lf*) mechanisms have been involved in C9ORF72 related ALS/FTLD. The *gf* mechanism of C9ORF72 has been studied in various animal models but not in *C. elegans*. In the present study, we described mutant C9ORF72 modeling in *C. elegans* and report the finding of two suppressor genes. We made transgenes containing 9 or 29 repeats of GGGGCC in C9ORF72, driven by either the *hsp-16* promoters or the *unc-119* promoter. Transgenic worms were made to carry such transgenes. Phenotypic analysis of those animals revealed that *P*_*hsp*−*16*_*::(G4C2)*_*29*_*::GFP* transgenic animals (EAB 135) displayed severe paralysis by the second day of adulthood, followed by lethality, which phenotypes were less severe in *P*_*hsp*−*16*_*::(G4C2)*_*9*_*::GFP* transgenic animals (EAB242), and absent in control strains expressing empty vectors. Suppressor genes of this locomotor phenotype were pursued by introducing mutations with ethyl methanesulfonate in EAB135, screening mutant strains that moved faster than EAB135 by a food-ring assay, identifying mutations by whole-genome sequencing and testing the underlying mechanism of the suppressor genes either by employing RNA interference studies or *C. elegans* genetics. Three mutant strains, EAB164, EAB165 and EAB167, were identified. Eight suppressor genes carrying nonsense/canonical splicing site mutations were confirmed, among which a nonsense mutation of F57A10.2/VAMP was found in all three mutant strains, and a nonsense mutation of acp-4/ACP2 was only found in EAB164. Knock down/out of those two genes in EAB135 animals by feeding RNAi/introducing a known acp-4 null allele phenocopied the suppression of the C9ORF72 variant related movement defect in the mutant strains. Translational conformation in a mammalian system is required, but our worm data suggest that altering acp-4/ACP2 encoding lysosomal acid phosphatase may provide a potential therapeutic method of reducing acp-4/ACP2 levels, as opposed or complementary to directly reducing C9ORF72, to relieve C9ORF72-ALS phenotypes. It also suggests that the C9ORF72-ALS/FTLD may share a pathophysiologic mechanism with vesicle-associated membrane protein-associated protein B, a homolog of F57A10.2/VAMP, which is a proven ALS8 gene.

## Introduction

Abnormal expansion of a GGGGCC hexanucleotide (G4C2) repeat in a noncoding region of C9ORF72 is the most commonly identified genetic variant associated with both familial amyotrophic lateral sclerosis (ALS) and familial frontotemporal dementia (FTD) (DeJesus-Hernandez et al., 2011; Renton et al., 2011). Various genetic models, including C9ORF72 knock-outs (KO) in *M. musculus* and zebrafish, have been constructed based on this finding (Ciura et al., 2013; O'Rourke et al., 2016). C9ORF72 *gain-of-function (gf)* models, testing Repeat-Associated Non-ATG Translation (RANT) of G4C2, have also been developed in *S. cerevisiae*, human iPSC-derived neuronal cells and *Drosophila*, which have identified nucleocytoplasmic transportation pathway anomalies as part of the pathogenetic mechanism of C9ORF72 associated ALS/FTLD (C9-ALS) (Mizielinska et al., 2014; Wen et al., 2014; Freibaum et al., 2015; Jovičić et al., 2015).

*C. elegans* offers a range of unique experimental advantages, many of which are not available in other organisms. Importantly, *C. elegans* has a fully developed nervous system and has most of the neurotransmitters that have been implicated in the pathogenesis of human neurologic illnesses, including the two major mammalian neurotransmitters, GABA and glutamate, as well as dopamine, serotonin, and acetylcholine neurotransmitter systems. Many gene systems of interest in neurology are conserved between *C. elegans* and humans, including those for neurotransmitter receptors, second messenger systems, ion channels, synaptic proteins, and enzymes required for neurotransmitter synthesis (Shaye and Greenwald, 2011). For example, ~83% of the *C. elegans* proteome has homologous genes in humans (Lai et al., 2000). In past studies, our group has shown that reverse genetic approaches in *C. elegans* identify important signal transduction pathways through which neurologic disease associated genes act (Buttner et al., 2007; Wang et al., 2016) and that pharmacogenomic studies in *C. elegans* identify novel drug targets relevant to neuropsychiatric disease (Karmacharya et al., 2009; Saur et al., 2013; Haeusler et al., 2014). Moreover, *C. elegans* reverse genetic experiments have defined novel mechanisms of action of FTD genes, specifically including the role of the programmed cell death pathway in the action of progranulin gene mutations (Kao et al., 2011). In *C. elegans*, a C9ORF72 homolog KO model was used to study the *loss-of-function* (*lf*) mechanism of C9ORF72 (Therrien et al., 2013). We also reported the observation of developmental and locomotive phenotypes based on C9ORD72/*alfa-1 lf* approaches (Wang and Buttner, 2013), but no such model has been described to test the toxic effects of the G4C2 disease-related fragment, thought to be an underlying pathophysiologic mechanism of ALS. Given that *C9ORF72*-linked neurodegeneration involves multiple *gf* mechanisms, including disrupted RNA homeostasis, toxic RNA foci and protein aggregates due to RANT (Ciura et al., 2013; Mori et al., 2013), there is potential value in defining the genetic pathways through which *C9ORF72* mutations produce phenotypic abnormalities in a *gf C. elegans* model. Here we report such C9ORF72 *gf* modeling in *C. elegans* and the subsequent findings of molecular components related to its ALS-like phenotypes. To the best of our knowledge, this is the first report of *C. elegans* C9ORF72 *gf* modeling of AS/FTLD.

## Materials and methods

### Nematode culture and strains

The nematodes were cultured under standard culture conditions in a 20°C incubator (Brenner, 1974). *C. elegans Bristol* strain N2 was used as a control. Other strains used/made are presented in Supplementary Table 1. Newly generated strains were backcrossed at least 4 times to the N2 strain. Nonsense and canonical splicing site mutations detected by next generation sequencing were confirmed by DNA Sanger sequencing at the Dana-Farber/Harvard Cancer Center DNA Resource Core.

### Plasmid constructions

To produce *Punc-119::(G4C2)*_*n*_ *::GFP* and *Phsp-16::(G4C2)*_*n*_*::GFP C. elegans* expression constructs, we first amplified human C9ORF72 genomic fragment containing the G4C2 repeat: 5′caaggagggaaacaaccgcagcctgtagcaagctctggaactcaggagtcgcgcgcta(ggggcc)_n_ggggcgtggtcggggcgggcccgggggcgggcccggggcggggctgcggttgcggtgcctgc3′. Primers for this reaction were available on request. Healthy human genomic DNA was used as PCR template. The PCR products were subcloned into the TOPO® TA Cloning vector (Invitrogen, CA USA) to get *(G4C2)*_*n*_ TOPO constructs, and then sequenced to determine the repeat number of G4C2. The maximal number we could achieve by this method was 29 due to the high GC% of the repeat sequence. The reference repeat number was determined as 9. (G4C2)_n_ TOPO constructs were digested with NotI and KpnI restriction enzymes to generate the fragment containing (G4C2)_n_, which was ligated with Fire Lab vectors L3787 and L3788 (Addgene) and processed with EagI and KpnI restriction enzymes. By this method, we generated *Phsp-16::(G4C2)*_*n*_*::GFP C. elegans* expression constructs (*n* = 29 or 9). The *Punc119::GFP C. elegans* expression construct, based on ppd95.75, was a gift from Prof. Craig Hunter at the Harvard Medical School. We inserted *(G4C2)*_*n*_ fragments from *(G4C2)*_*n*_ TOPO constructs into *Punc119::GFP* constructs using XbaI and KpnI restriction enzymes to generate *Phsp-16::(G4C2)*_*n*_*::GFP C. elegans* expression constructs (*n* = 29 or 9). All plasmid constructions were confirmed by sequencing at the Dana-Farber/Harvard Cancer Center DNA Resource Core.

### Transgenic animals

Extrachromosomal transgenic lines were produced by injecting *Punc-119::(G4C2)*_*n*_*::GFP* and *Phsp-16::(G4C2)*_*n*_*::GFP*constructs at 40~50 ng/μl into young adult *N2* gonads (Mello et al., 1991). L3787, L3788 and *Punc119::GFP* constructs were also injected at 40~50 ng/μl into N2 gonads to generate the control worm strains. *Pmyo-3::DsRed2* (pHC183) at ~50 ng/μl was used as a co-injection marker (Saur et al., 2013). 2–3 independent extachromasomal lines per construct were integrated into the germline by a standard gamma radiation method described by Prof. Michael Koelle at Yale University. The gamma radiation experiments were performed at Prof. Mark Alkema's laboratory at UMASS Medical School. Two independent lines per construct were outcrossed to N2 strain at least 4 times. Only experimental results that were consistent across both lines per construct were reported. A list of the transgenic animals we made appears in Supplementary Table 1. To obtain imaging, worms were picked onto a slide with an agarose pad containing 20 mM sodium azide, and observed under a Zeiss Axio2 microscope (Leica).

### Screening for suppressors of the movement defect of EAB135 *[Phsp-16::(G4C2)_*29*_::GFP]*

We selected EAB135 animals and took advantage of their movement defects to perform genetic suppressor screens. We adopted the principle of the food-ring assay (Zengel and Epstein, 1980). Animals of L4 progeny (P0) were harvested and treated with ethyl methanesulfonate (EMS), after rinsing off EMS with M9 buffer, those animals were cultured at 20°C for 36–48 h until they reached the second day of adulthood, then they were bleached to obtain synchronized L1s (F1) after overnight incubation. F1s were cultured to the first day of adulthood and were bleached to obtain synchronized L1s (F2). NGM plates of 100 cm diameter were seeded with OP50 on the edge of the surface and were incubated at 37°C overnight to form a bacterium ring 1 day prior to the addition of F2s grown to Day 1 adults. Those animals were rinsed multiple times with M9 buffer until they were free of bacteria, and were placed in the center of the plates. Animals reaching the ring faster than the original EAB135 animals were picked as suppressor candidates. This procedure usually generated about 3–5 candidates for each round of screens of ~16,000 haploid genomes, and we performed five rounds of screens.

### Whole-genome sequencing and data analysis

Synchronized L1 animals were seeded on 10 NMG plates (8.5 cm diameter), grown until they were gravid adults, and then washed off with M9 buffer and harvested in several 50 ml Conical tubes. After rinsing with M9 2–3 times, the animals were left on a NUTATOR mixer for 2 h to eliminate food in the gut, then were washed again with M9 2–3 times. The supernatant was removed after centrifugation at 1500 g for 3 min. A total 500 μl worm pellet was achieved by such method. For DNA extraction, we followed the protocol of Gentra Puregene Kit (Qiagen). The concentration of the final DNA preparation was determined by the Qubit assay (Invitrogen) at the Bauer Core Facility, Harvard University. 1 μg DNA was used for further sequencing library construction and next generation sequencing at BGI Americas (Cambridge MA USA). In brief, the sequencing libraries were made per the TruSeq®DNA PCR-free sample preparation kit (Illumina), and next generation sequencing was performed on an Illumina HiSeq seq platform. The sequencing results were analyzed both at BGI Americas Inc. and at the High-performance Computation Facility of the Partners Healthcare System. In brief, the sequencing reads were first mapped to the reference genome, such as WS220, with Bowtie-0.12.7, the variants were called with Samtools, and they were visualized by the Integrative Genomics Viewer developed by the Broad Institute. Nonsense mutations producing nonsense-mediated mRNA decay (NMD) and canonical splicing site mutations were noticed and confirmed by Sanger sequencing.

### Feeding RNAi

We employed gene-specific RNAi cultures from Ahringer's RNAi feeding library (Geneservice Ltd) to carry out RNAi experiments, using the Ahringer's feeding RNAi protocol (Kamath, 2000). RNAi exposed cultures were grown overnight at 37°C in 100 ml of LB with 100 mg/ml of ampicillin, then poured on NGM plates containing 25 μg/ml carbenicillin and 1mM IPTG. The cultures were dried and induced overnight in a 37°C incubator. Animals cultured on OP50 were collected and bleached to obtain embryos, and were starved in M9 buffer to obtain synchronized L1s. Those L1 stage animals were then cultured on NGM plates seeded with HT115 to achieve the desired stage for RNAi. We used *dpy-6* RNAi as the positive control.

### Developmental/behavioral assays

To obtain the developmental data, we bleached the well-grown adult animals using standard bleach solution to obtain synchronized L1s, 25~50 of which were then placed on seeded NGM plates. Afterwards, the plates were observed and the developmental status was recorded every 24 h for 5–10 days. At least three independent experiments were performed for each study and a typical result was shown. For thrashing assays, we minimized contamination with *E. coli* by rinsing and soaking animals in two Petri dishes filled with M9 buffer. Animals were then transferred to NGM plates (35 mm diameter) with 1 ml of M9 buffer and allowed to accommodate for 1 min. We counted the number of thrashes for 20 s under the dissecting microscope. Only continuous movements were scored, and at least 25 animals were tested for each condition.

### Statistical analysis

Data were expressed as means ± standard deviations. Statistical comparisons were all performed using unpaired, two-tailed Student's *t*-tests.

## Results

### Phenotypic characterization of *(G4C2)_*9/29*_*::GFP strains

**Figure 1:**
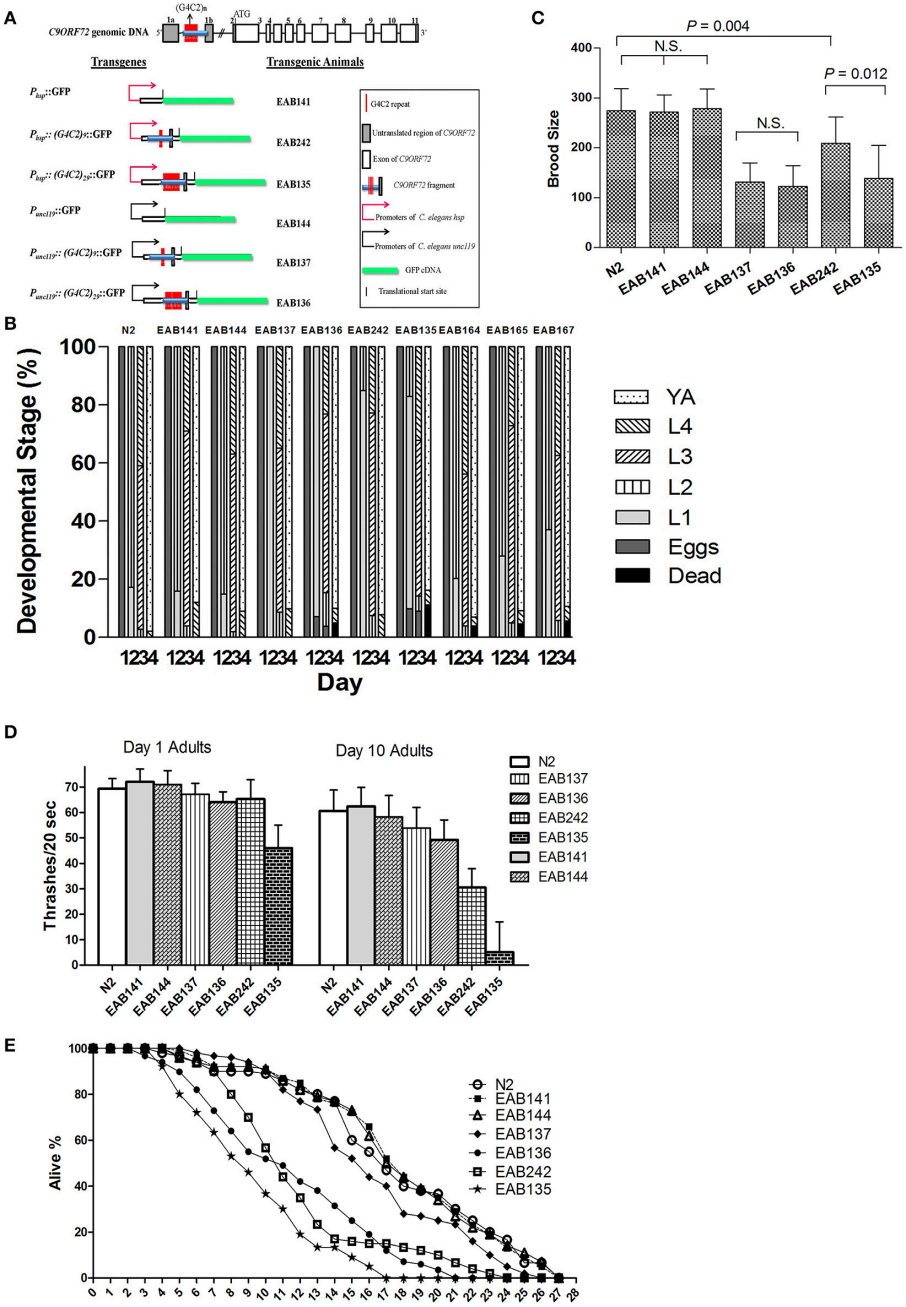
**A forward genetic screen identified F57A10.2/VAMP and acp-4/ACP2 as suppressors of C9ORF72 pathology in ***C. elegans*****. Generally *(G4C2)*_*29*_ or *P*_*hsp*_ animals produced more severe deficit phenotypes than *(G4C2)*_*9*_ or *P*_*unc119*_, except for the brood size assay. EAB164 animals carrying both mutations of F57A10.2/VAMP and acp-4/ACP2 had the most suppression of all tested phenotypes. *Student t*-tests were performed to generate *P*-values. **(A)** Transgenes and transgenic *C. elegans* lines. **(B)** Animals expressing C9ORF72 disease-related fragments had delayed developmental phenotypes and suppressors from the screen partially relieved the defect. N2 (wide type), EAB141, EAB144, EAB137, EAB136, EAB242, and EAB135, and suppressors, EAB164, EAB165, and EAB167 were staged for 4 days from about 50 eggs/strain at room temperature. At least three experiments were performed in each case, and a typical experimental result is shown. For the *P*_*hsp*_ animals, EAB135 had the highest lethality on Day 4, compared to strain EAB 242, and both had higher lethality than EAB141 and N2 animals. The lethality was partially relieved in EAB164, EAB165, and EAB167. A similar trend was seen in *P*_*unc*119_animals. **(C)** Transgenic animals displayed reduced brood sizes. EAB135, EAB136, and EAB137 animals had similar degrees of reduced brood sizes compared to N2, EAB141, and EAB144 animals. For example, EAB135 and EAB136 strains had 139 and 123 brood sizes, compared to 272 and 279 in EAB141 and EAB144 strains, respectively. At least 15 animals per strain were scored in a single experiment, and three independent experiments were performed. **(D)** Transgenic animals showed age-dependent locomotion defects in swimming assays. Thrashes/20 s were recorded. Statistically significant differences were found between and among strains, as below, Day 1: EAB135 vs. EAB242 (46 vs. 65, *P* < 0.05); Day 10: both EAB135 and EAB242 vs. EAB141 (5 and 31 vs. 62, *P* < 0.05 and < 0.001, respectively). Three independent experiments were performed in each case. In each experiment, about 30 animals per strain were scored. **(E)** Transgenic strain EAB135 had the most severely impaired life span, followed by EAB136 and EAB242 animals. EAB141, EAB144, and EAB137 had a life span similar to N2 animals. Three experiments were independently performed in each case. Results of a typical experiment are shown, using 35 animals per strain, in which EAB135 animals had a reduced life span of 17 days, compared to about 26 days for the EAB141 strain.

**Figure 2:**
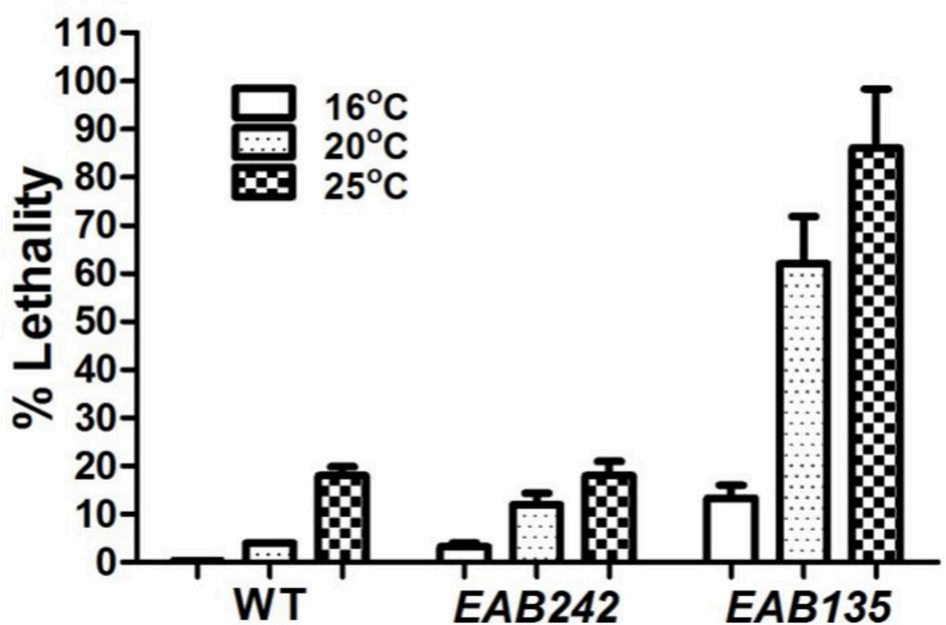
**The ***(G4C2)***_***29***_ transgene is toxic**. Percent lethality on day 10 of adulthood at three different temperatures are shown. The wild-type N2 (WT) strain displayed little or no lethality at any temperature tested. Strain EAB135, carrying the *(G4C2)*_*29*_ transgene, displayed much greater lethality at 20°C or 25°C than strain EAB242, carrying the *(G4C2)*_*9*_ transgene (both *P* < 0.01).

**Figure 3:**
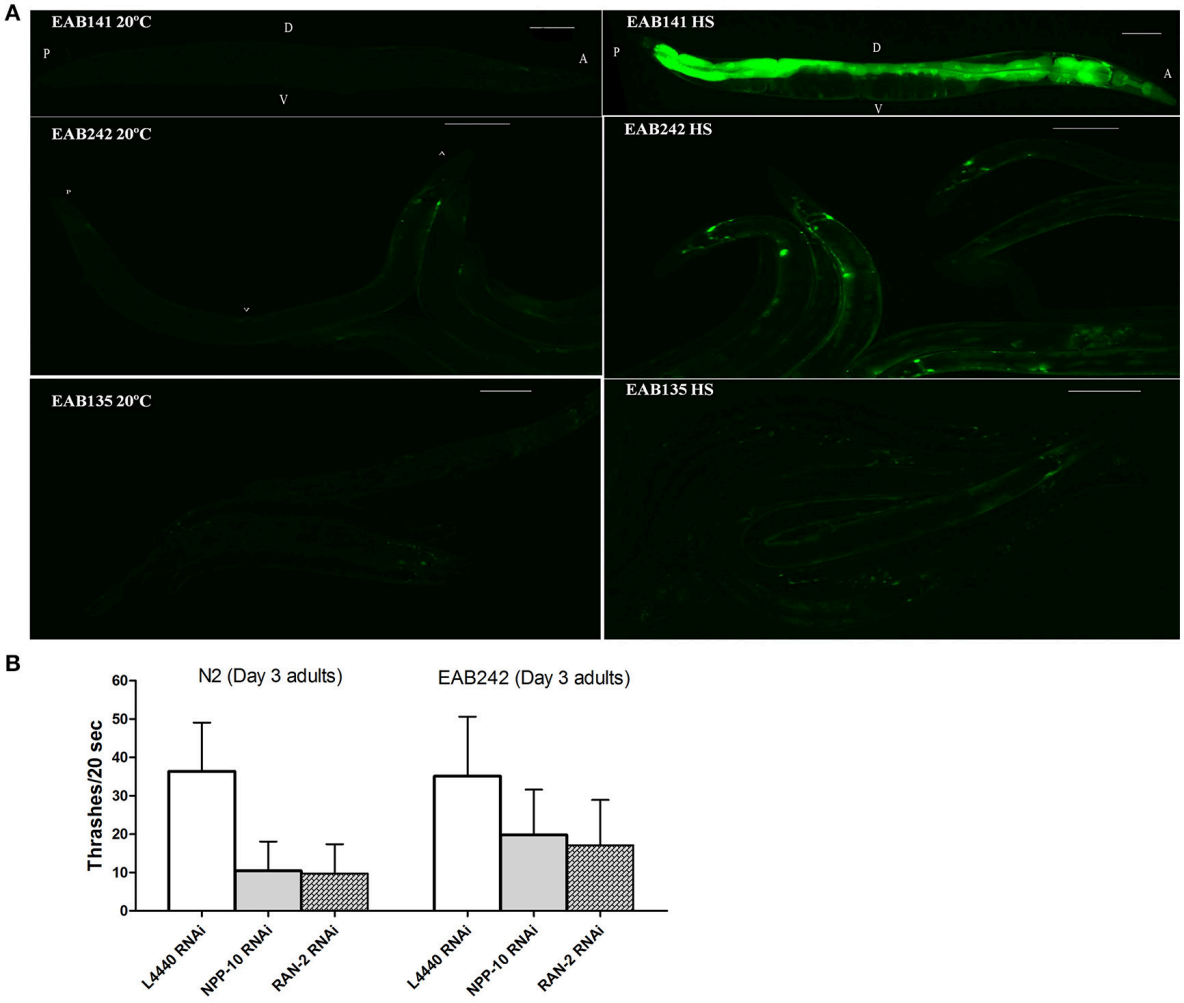
**The nuclearpore transportation (NPT) pathway might be impaired in ***P***_***hsp***−***16***_***::(G4C2)***_***9/29***_***::GFP*** transgenic animals. (A)** Heat shock-driven expression of *P*_*hsp*−*16*_*::GFP* transgenes. Animals were cultured at 20°C and then exposed to 31°C heat shock for 1 h (right panels) or to no heat shock (left panels). GFP was significantly expressed after heat shock in EAB141 animals, but was not much enhanced in EAB242 and EAB135 animals. Transgenic constructs were co-injected with a *Pmyo-3::DsRed2* marker. Scale bars are 100 μm. **(B)** At the L4 stage, N2 and EAB242 animals were treated with NPP-10/NUP98 RNAi and RAN-2 RNAi for 72 h. NPP-10 RNAi reduced thrashes/20 s from 36.3 to 10.4 in N2s and from 35.1 to 19.8 in EAB242s (%Changes: 71.3% vs. 43.6%). RAN-2 RNAi reduced the thrashes to 9.7 in N2s and 17.1 in EAB242s (%Changes: 73.3% vs. 51.3%). L4440 control RNAi did not affect thrashes (36.3 and 35.1 of N2 and EAB242, respectively).

Transgenes were driven by either the *hsp-16* promoter, for conditional global expression, or by the *unc-119* promoter, for pan-neuronal expression. C9ORF72 genomic fragments of *(G4C2)*_*9/29*_ in those transgenes were inserted between the transcriptional start site and the translational start site, thus mimicking their role as part of 5′-UTR in human C9ORF72 (Figure 1A). Integrated transgenic animals were obtained. At 25°C, *P*_*hsp*−*16*_*::(G4C2)*_*29*_*::GFP* transgenic animals (strain EAB 135) displayed severe paralysis by the second day of adulthood, followed by lethality. These phenotypes were not observed in wild-type animals and animals expressing the empty vector (strain EAB141), and were less severe in animals carrying the *P*_*hsp*−*16*_*::(G4C2)*_*9*_*::GFP* transgene (strain EAB242). For example, ~85% of EAB135 animals were dead by day 10 of adulthood when cultured at 25°C, compared to ~18% for N2 or EAB242 (Figures 1D, 2). When culturing at 20°C without heat-shocking, EAB135 animals still showed a number of age-dependent phenotypes, including increased lethality, movement defects, reduced brood size and impaired life span (Figures 1B–E). In the swimming assay, EAB135 animals had 45 and 4.5 thrashes/20 s at Day 1 of adulthood and at Day 10 of adulthood, respectively; both were significantly compromised relative to 71 and 60.7 thrashes/20 s in EAB141 animals carrying the empty vector (*P* < 0.05 and < 0.001, respectively. See Figure 1D). EAB 242 animals had similar but less severe movement deficits and reduced life span phenotypes, compared to the normal behaviors and life span of strain EAB141. At Day 10 of adulthood, EAB242 animals had 30.5 thrashes/20 s, which fell in the middle of EAB141 control animals and EAB135 animals. EAB242 animals also had a life span of about 24 days between 17 days of EAB135 and 28 days of EAB141. To our surprise, both EAB243 and EAB135 animals did not show significant enhancement of GFP when exposed to various heat shock protocols, which response was intact in those strains expressing the empty vector (Figure 3A). We confirmed the presence of the transgenes in EAB135 and EAB242 animals by PCR and sequencing. Given the repeat number dependent phenotypes mentioned above and that the heat shock response of *hsp-16.2/41* promoters requires the nuclearpore transportation (NPT) pathway (Rohner et al., 2013), which is impaired in C9ORF72-ALS/FTLD (Wen et al., 2014; Freibaum et al., 2015; Jovičić et al., 2015), it appears possible that this *loss-of-heat shock* response is due to G4C2 repeats, as well. If that is the case, interrupting the NPT pathway would likely result in a more devastating effect to wide type animals, rather than to EAB135/EAB164 animals, in which that pathway has already been impaired. Indeed, when we reduced the levels of NPP-10 (ortholog of human Nucleoporin, NUP98) or RAN-2 (Ran GTPase activating protein homolog), two conserved components of the NPC pathway, the movements of N2 animals were more severely affected than those of EAB242 animals after treatment with RNAi for 72 h from the L4 stage, as shown in Figure 3B. Knocking down NPP-10 reduced thrashes/20 seconds from 36.3 to 10.4 in N2s and from 35.1 to 19.8 in EAB242s (%Changes: 71.3% vs. 43.6%). Similarly, RAN-2 RNAi lowered the thrashes to 9.7 in N2s and 17.1 in EAB242s (%Changes: 73.3% vs. 51.3%). Meanwhile, both strains treated with L4440 control RNAi showed a similar number of thrashes (36.3 and 35.1 of N2 and EAB242, respectively). We also tried other NPT components, such as NPP-5/NUP107 and NPP-6/NUP160 RNAi, but both N2s and EAB242s were too sick to perform in further experiments after 1 day of RNAi exposure. We did not use EAB135 animals as their significantly impaired movement could be noticed as early as the L4 stage, as compared to N2 animals, which would make any results difficult to interpret. Collectively, we concluded that the NPT pathway is likely to be impaired in our EAB242 animals, which supports the interpretation that the impaired heat shock response observed in these animals is due to G4C2 repeats. We would emphasize that several head neurons (unidentified) of EAB242 and EAB135 were visible under the regular dissecting microscope at 22~25°C. We monitored both this neuronal GFP signal and the injection marker, a muscular Ds-RED fusion, when selecting integrated lines. All of the integrated lines of *P*_*hsp*−*16*_*::(G4C2)*_*9/29*_*::GFP* carried both signals with a 100% transmission rate. Thus, the possibility that the transgenes were not integrated into the genomes is quite low. In summary, those transgenic animals with global expression of the disease-associated construct displayed a series of repeat-number dependent phenotypes, among which, movement defects and compromised life span resemble some of the features of illness observed in ALS patients.

How might neuronal expression of *(G4C2)*_*9/29*_ play a role in producing those phenotypes? Most of the above-mentioned phenotypes were also observed in the pan-neuronal expressional strains of G4C2 [EAB137: *(G4C2)*_*9*_, EAB136: *(G4C2)*_*29*_], but abnormalities were relatively less severe compared to those in the globally expressed versions. Albeit, the promoters had to be different between those transgene constructs. For example, in the swimming assay, Day 10 adults of EAB136 had 45.7 thrashes/20 s, which was much improved when compared to 4.5 thrashes of EAB135 animals (Figure 1D). Almost all the tested phenotypes of the pan-neuronal transgenic displayed a trend toward G4C2 repeat number dependency with the exception of brood size. Both EAB136 and EAB137 showed a similar reduction of brood size, which was comparable to EAB135 animals in the normal culturing condition. This may be a floor effect induced by the pan-neuronal transgenes, but further studies would be needed to validate this possibility.

Collectively, we created a series *C. elegans* strains carrying disease-related genomic fragments of C9ORF72, and we observed a sequence-number-repeat/age-dependent locomotor phenotype, which might be useful for an unbiased genetic screen.

### Screening for suppressors of locomotive defects of EAB135

We initially studied the EAB136 animals, as the pan-neuronal expression of the transgene might make it a better targeted model for human ALS-FTLD. However, in the pilot experiments we failed to distinguish EAB136s and N2s at Days 2–5, by directly observing their movements under the dissecting microscope, as the locomotor defect of EAB136 was too subtle. We thus chose to study EAB135 animals, the global expressional version, for a genetic suppressor screen based on locomotion. We performed the screen as described in Materials and Methods.

**Figure 4:**
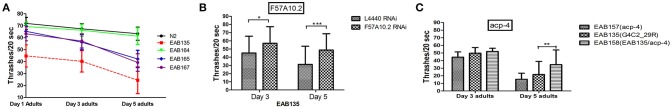
**Suppressor genes F57A10.2 and acp-4 may exert their effects on C9ORF72 associated pathology via a ***loss-of-function*** mechanism. (A)** EAB164, EAB165, and EAB167 were identified as suppressors of the slow movement phenotype of EAB135. All three suppressors were associated with improved swimming activity in thrashing assays using Day 1, Day 3, and Day 5 adults, compared to EAB135 animals. Strain EAB164 was the most significantly improved suppressor strain, showing almost similar movement ability to N2 animals. **(B)** Downregulation of suppressor gene F57A10.2 in EAB135 animals, by feeding RNAi, produced improved movement in swimming assays, with swimming resembling activity in strains carrying the potential relative null alleles. Total numbers of 50–98 animals were used in each condition. Three experiments were performed in each case. ^*^P < 0.05, ^**^P < 0.0002, ^***^P < 0.00001. **(C)** We constructed EAB158 carrying acp-4 (*lf,gk833833*) on EAB135 background. EAB158 animals moved significantly better than both EAB135 and EAB157 (*gk833833*). Total numbers of 75–98 animals were used in each condition. Three experiments were performed at room temperature in each case. ^**^P < 0.0002.

Overall we identified 27 candidates using Day 1 adulthood animals. Then we performed 6-9 validations. Only those passing all the validations were studied further. 24 of the 27 suppressor strains failed validations at least twice. The remaining three suppressors passed all 9 validations and were named EAB164, EAB165 and EAB167. EAB164 is a stronger suppressor, in terms of movement, developmental measures (Figures 1E,D, 4A) and life span (data not shown), than the EAB165 and EAB167 strains. For example, at Day 5 of adulthood, N2 animals and EAB135 animals had 63.3 and 24.4 thrashes/20 s, respectively. EAB164 behaved similarly to N2 and had 61.3 thrashes/20 s. The other two suppressors moved slower than N2 and EAB164, but still faster than EAB135. Their thrashes/20 s were 42.2 (EAB165) and 39.2 (EAB166). Genome-wide next generation sequencing was performed to identify genetic variants unique to the three suppressors. Each strain carried at least two nonsense mutations or canonical splicing site mutations (see Table 1): EAB164 animals carry mutations of acp-4, ugt-42 and F57A10.2; EAB165 animals carry mutations of nhr-181 and F57A10.2; EAB167 animals carry T10E9.2, str-46, F57A10.2, dnj-25 and mrp-2 mutations. Notably, the same nonsense variant of F57A10.2 was identified in all three suppressors, but not in wild type animals or EAB135 animals. That nonsense variant would probably induce NMD, given it localizes more than 55 nucleotides of the end of the second-to-last exon. Even if it escaped NMD, the resultant 76 amino acid truncated F57A10.2 protein would likely destroy the conserved human sperm protein (HSP) domain. Thus, it is probably an F57A10.2 null mutant. The HSP domain is shared with human VAMPs, including VAPA and VAPB. Interestingly, two pathogenic mutations within the HSP domain encoding the sequence of VAPB, P56S, and T46I, have been identified in ALS8 patients (Nishimura et al., 2005; Chen et al., 2010). Evidence of disruption of normal *VAPB* expression, formation of intracellular aggregates and impairment of axonal transportation of mitochondria, all suggest that these two variants exert dominant negative functions in ALS8(Teuling et al., 2007; Chen et al., 2010; Mórotz et al., 2012). This is consistent with our results that the “null” of the *C. elegans* homolog of the human gene, F57A10.2, reversed the ALS-related phenotypes. This possibility is further supported by the observation that reduction of F57A10.2 mRNAs leads to improved movement when EAB135 animals are exposed to F58A10.2 RNAi bacteria. For example, N2 animals treated for 5 days with F57A10.2 RNAi bacteria had about 48.7 thrashes/ 20 s, which was significantly more than the 31.2 thrashes/20 s of those treated with L4440 control RNAi bacteria (Figure 4B). The other confirmed nonsense variant is in acp-4 of the EAB164 strain. That variant may also induce NMD as it is on the fourth last exon from the 3′ end of the gene. Thus, it is also presumably a null allele. We obtained the VC40826 strain carrying a known acp-4 null allele (*gk833833*: nonsenseR290Opal) from CGC. VC40826 animals had very obvious movement defects, a phenotype we used for out-crossing with N2 animals, for five times, to get the EAB157 strain. EAB157 still displayed movement defects and we confirmed *gk833833* in EAB157 by PCR-sequencing. We crossed this strain with EAB135 animals in order to introduce *gk833833* into the EAB135 background, and generated strain EAB158 [acp-4(*gk833833*); EAB135 (*Phsp16::(G4C2)*_*29*_*::GFP*)]. Consistent with the *lf* hypothesis, EAB158 animals had an improved movement phenotype compared to both EAB135 and EAB157, based on their Day 5 data. On Day 3, a substantial difference did not exist (Figure 4C). Meanwhile five other genes harboring nonsense variants or splicing site mutations were also tested for an *lf* mechanism, but RNAi experiments based on EAB135 animals failed to reveal improvement of movement (data not shown), which indicates either that those variants are not disease-causal or that they may not act through an *lf* mechanism.

**Table 1:** **List of ***C. elegans*** genes that carry significant mutations in the EAB135 suppressors identified from the screen based on slow movement**.

***C. elegans* gene/chromosome**	**Human ortholog/homolog**	**Protein and function**	**Position**	**Codon change**	**Transcritional/translational prediction**
**EAB164**					
acp-4/II	ACP2, ACPT, ACPP	Lysosomal acid phosphatase activity	8434143	CGA>TGA	STOP
ugt-42/V	UGT3A1,UGT3A2	Transferase activity, transferring hexosyl groups	648416	GT>AT	TAA introduced
F57A10.2/V	VAPA, VAPB		15766903	TCA>TGA	STOP
**EAB165**					
nhr-181/V	HNF4G	A putative nuclear hormone receptor (NHR)	4097868	AG>AA^*^	Frame shift
F57A10.2/V	VAPA, VAPB		15766903	TCA>TGA	STOP
**EAB167**					
T10E9.2/I			6543341	TCA>TAA	STOP
str-46/V		7 transmembrane GPCR, serpentine receptor class r (Str)	2899516	TGG>TGA	STOP
F57A10.2/V	VAPA, VAPB		15766903	TCA>TGA	STOP
dnj-25/V	auxilin	Required for clathrin-mediated endocytosis and for normal development	20743985	AG>AT^*^	PE deletion, frame might not shift
mrp-2/X	MRP1, MRP2	A predicted multidomain transmembrane protein that is a member of the ATP-binding cassette superfamily of transport proteins	569906	TGG>TGA	STOP

* *Splicing site. ACPT, acid phosphatase, testicular; ACPP, acid phosphatase, prostate; ACP2, acid phosphatase 2, lysosomal; UGT3A1, UDP glycosyltransferase 3 family, polypeptide A1; UGT3A2, UDP glycosyltransferase 3 family, polypeptide A2; VAPA/B, vesicle-associated membrane protein-associated protein A/B; HNF4G, isoform 2 of hepatocyte nuclear factor 4-gamma; MRP1, multidrug resistance-associated protein 1; Dnj-25 is a potential model for Parkinson's disease*.

## Discussion

The genetic interaction between F57A10.2/VAPB and the disease-related repeat of C9ORF72 suggests that F57A10.2/VAPB is required in C9ORF72-pathology. C9ORF72 has been demonstrated to participate functionally in endosomal trafficking, and it co-localizes with Rab proteins (Farg et al., 2014). C9ORF72 itself is structurally related to DENN Rab-GEFs (Levine et al., 2013). Additionally, fly VAPB VAP has been functionally linked to rab-5 (Sanhueza et al., 2015). Thus, it is conceivable that *lf* of F57A10.2/VAPB underlies the toxic effects of C9ORF72 repeats through a RAB pathway. Caution needs to be noted that the effects of *(G4C2)* repeats may be irrelevant to the normal function of C9ORF72.

The stronger suppression of C9ORF72-phenotypes in EAB149 animals carrying mutations of both F57A10.2 and acp-4 suggests that those two suppressor genes may belong to different pathways; that their effects may be additive. That deletion of acp-4/ACP2, which probably encodes lysosomal acid phosphatase, suppressed the C9ORF72 phenotypes suggests that over-activated lysosomal activity might be pathogenic in ALS-FTLD. A number of reports have described elevation of autophagy/lysosomal activity in various tissues from ALS models/patients (Baker et al., 2015; Pagliardini et al., 2015). Reducing autophagy activation by n-butylidenephthalide (n-BP) improved symptoms in ALS transgenic mice (Hsueh et al., 2016) and upregulating autophagy function, by rapamycin, an autophagy enhancer, worsened survival of the ALS transgenic mice (Zhang et al., 2011). Therefore, down-regulation of the autophagy-lysosome pathway activity via lysosomal ACPs may represent a potential therapeutic target. Interestingly, C9ORF72 may rescue defects resulting from impaired lysosomal pathway activity in cultured cells (Busch et al., 2016). These interactions tentatively position C9ORF72 as a two-way regulator of the lysosomal pathway. Again, by way of caution, *lf* of ACP2 may result in cerebella degeneration/malformation and even neonatal death (Mannan et al., 2004), which indicates that a proper, intermediate, level of ACP2 needs to be achieved. Also, before this target could be used for therapeutic intervention, it would be essential to determine exactly what reduction of acp2 in which specific cell/tissue would be sufficient to produce improvement (Corbier and Sellier, 2016).

A C9ORF72 *lf* mechanism has been evidenced in the pathogenetics of C9ORF72-linked ALS-FTD, as well (O'Rourke et al., 2016). Our group has developed a C9ORF72/ *alfa-1(lf)* model in *C. elegans* (EAB129: *gk498021* of *alfa-1*). RNA-seq experiments identified a series of differentially expressed genes in that *alfa-1(lf)* animal and N2s (unpublished data). One of the differentially expressed genes, *ZK370.8*, is an ortholog of human TTC1 (tetratricopeptide repeat domain 1). Removal of TTC1 produces abnormal vesicular transport in human cells (Lotz et al., 2008). TTC1 also forms a complex with HSP90 and Rab-8, and the latter has been suggested to be activated by C9ORF72 and to function together with C9ORF72 in mediating autophagy. With many missing pieces to this puzzle, our *C. elegans* models, both *lf* and *gf*, would no doubt exert more power in the future.

In summary, we developed a disease associated allelic C9ORF72 model of ALS/FTLD in *C. elegans*, then identified and confirmed two genes that harbor genetic variants which may rescue the disease gene related phenotypes. Those genetic modifiers might offer new insight on the mechanism by which the C9ORF72 mutation produces ALS/FTLD and suggest a target for potential therapeutic intervention.

## Author contributions

XW, EB, BC designed experiments and interpreted the results. XW, LH, TS, KJ, YZ, DZ, JL conducted the experiments. MP and GC provided vital reagents and contributed to data analysis. XW and BC wrote the paper.

## Funding

Some strains were provided by the CGC, which is funded by the NIH Office of Research Infrastructure Programs (P40 OD010440). The work was supported by an NIH Clinical Scientist Development award, K08NS002083 (EB), funds of the Program for Neuropsychiatric Research, at McLean Hospital (BC), National Natural Science Foundation grant 81400940 (XW) and a NARSAD Young Investigator Award to EB. BC is the Robertson-Steele Professor of Psychiatry at Harvard Medical School.

### Conflict of interest statement

The authors declare that the research was conducted in the absence of any commercial or financial relationships that could be construed as a potential conflict of interest.
